# Augmenting patient safety surveillance in radiation oncology with large language model-based root cause analysis: A proof-of-concept study

**DOI:** 10.1371/journal.pdig.0001740

**Published:** 2026-09-25

**Authors:** Yuntao Wang, Mariluz De Ornelas, Matthew T. Studenski, Elizabeth Bossart, Siamak P. Nejad-Davarani, Yunze Yang

**Affiliations:** Department of Radiation Oncology, University of Miami, Miami, Florida, United States of America; University of A Coruna: Universidade da Coruna, SPAIN

## Abstract

We investigated the potential utility of large language models (LLMs) in supporting patient safety efforts. Specifically, we evaluated the reasoning capabilities of LLMs in performing root cause analysis (RCA) of radiation oncology incidents using narrative reports from the Radiation Oncology Incident Learning System (RO-ILS). We prompted four state-of-the-art LLMs, Gemini 2.5 Pro, GPT-4o, o3, and Grok 3, with the “Background and Incident Overview” sections from 19 publicly available RO-ILS cases. Each model was instructed to perform RCA and generate root causes, lessons learned, and suggested actions using a standardized prompt based on AAPM RCA guidelines. Model outputs were evaluated using a combination of objective semantic similarity metrics (cosine similarity via Sentence Transformer), semi-subjective assessments (precision, recall, F1-score, expert-adjudicated PPV (Positive Predictive Value), hallucination rate and performance criteria including relevance, comprehensiveness, quality of justification and quality of solution), and subjective ratings (reasoning quality and overall performance) by five board-certified medical physicists. LLMs demonstrated satisfactory performance across evaluation metrics. All models exhibited some degree of hallucination, ranging from 11% to 61%. All the evaluated LLMs demonstrated comparable baseline capabilities in objective causal extraction, and Gemini 2.5 Pro exhibited the highest overall performance score among 4 models. Statistically significant differences were observed among models in expert-adjudicated PPV, hallucination rate, and subjective ratings (*p* < 0.05). LLMs delivered promising results as assistive tools for RCA in radiation oncology, with the ability to generate relevant and accurate analyses aligned with expert expectations. LLMs may support incident analysis and contribute to quality improvement efforts to advance patient safety in clinical radiation oncology practice.

## Author summary

Patient safety is paramount in radiation oncology treatment. This pilot study represents a pioneering investigation into the utility of Generative Artificial Intelligence (GAI) for enhancing critical safety mechanisms. Our research innovatively targets the sophisticated reasoning capabilities of Large Language Models (LLMs) in Root Cause Analysis (RCA), a complex, domain-specific process essential for preventing medical errors. We conducted a systematic evaluation of four advanced LLMs using real-world clinical incident reports to identify underlying systemic failures. Our results demonstrate that these AI models function as highly capable reasoning tools within clinical settings. All evaluated models received high validation from board-certified medical physicists. This innovation signals a paradigm shift in clinical implementation of digital health, proving that AI can successfully execute complex clinical reasoning tasks. By establishing a foundation for reliably augmenting human judgment in safety-critical domains, this proof-of-concept work provides a roadmap for integrating scalable, intelligent safety surveillance systems in radiation oncology, ultimately improving care for cancer patients.

## Introduction

The integration of generative artificial intelligence (GAI), particularly Large Language Models (LLMs), has rapidly expanded across the medical landscape, offering transformative solutions for tasks ranging from administrative automation to complex clinical decision support [1–3]. The transition of LLMs from advanced pattern recognition to architectures capable of emergent reasoning represents a fundamental breakthrough in GAI, driving a transformative leap in clinical medicine [4–6]. This progress stems from immense model scale and architectural innovation [7]. Models with trillions of parameters capture complex relationships from vast datasets [8], while architectures like Mixture-of-Experts (MoE) improve efficiency by selectively activating specialized sub-networks for specific tasks [9]. This is also cultivated through multiple approaches in training and prompting [10]. Training strategies, such as Supervised Fine-Tuning (SFT) [11] and Reinforcement Learning from Human Feedback (RLHF) [12,13], fundamentally align the model’s logic with human intent and coherence. In addition, dynamic prompting strategies like Chain-of-Thought (CoT) [14] and Tree-of-Thought (ToT) [15] are employed to structure the model’s deliberation, guiding it to break down complex problems and explore solutions step-by-step [16].

Such progression of reasoning capabilities is evidenced by their improving performance on a variety of complex tasks [17], including natural language understanding, code generation [18], and problem-solving [19,20]. The development of diverse benchmarks such as MMLU (Massive Multitask Language Understanding) [19], GPQA (A Challenging Benchmark for Advanced Reasoning) [21], and various coding and commonsense reasoning tests (e.g., HumanEval, HellaSwag) have been crucial in quantifying these advancements [19]. Models’ mathematical reasoning is evaluated on benchmarks ranging from grade-school problems (GSM8K) [20] to advanced competition math (MATH) [19]. Pushing this frontier, a recent Gemini model demonstrated capabilities competitive with the brightest human minds, achieving a gold-medal standard in the International Mathematical Olympiad (IMO) competition [22]. However, evaluating the reasoning capabilities of LLMs remains an ongoing challenge. Issues such as inherent biases learned from vast training datasets, the potential for “hallucination” or the generation of incorrect information, and the reproducibility and robustness of their reasoning processes are active areas of research [23,24]. The sensitivity of these models to different prompting strategies also complicates efforts to ensure their reliability and trustworthiness, a critical concern in safety-critical applications.

Radiation oncology is a highly complex medical discipline that integrates advanced technology, intricate clinical workflows, and multidisciplinary collaboration. The treatment process spans multiple stages, from imaging and contouring to treatment planning, quality assurance, and daily delivery, creating numerous interfaces where systemic failures or human errors can occur. Given the high doses of radiation involved, ensuring patient safety and treatment quality is paramount [25,26]. Radiation Oncology Incident Learning Systems (RO-ILS), managed by American Society for Radiation Oncology (ASTRO), enable confidential reporting, collection, and analysis of errors, near misses, and unsafe conditions with narrative texts [27]. The primary goal of RO-ILS is to foster a culture of safety through shared learning from these incidents to prevent future occurrences [28,29]. A critical component of analyzing reported incidents is Root Cause Analysis (RCA) [30,31]. Current RCA methodologies in radiation oncology, often guided by American Association of Physicists in Medicine (AAPM) recommendations and adapted from broader healthcare and industrial safety practices, typically involve a systematic process. This process includes forming a multidisciplinary team, gathering data surrounding the event, creating a sequence or timeline of events, repeatedly asking “whys” to drill down to fundamental causes, identifying contributing factors, and formulating corrective and preventive actions. This structured, human-driven analytical process is essential for understanding the complex interplay of factors that can lead to incidents in the radiation therapy workflow.

The intersection of rapidly advancing reasoning capabilities of LLMs and the critical need for robust safety analyses in specialized domains like radiation oncology presents a compelling area of investigation. This paper seeks to perform RCA of radiation oncology incidents using LLMs and to evaluate their reasoning capabilities within this highly specific and safety-critical domain. This task is uniquely challenging as it demands not only a nuanced understanding of domain-specific knowledge, encompassing clinical procedures, medical physics, radiation therapy equipment, and quality assurance protocols, but also sophisticated reasoning abilities. These abilities include identifying causal relationships, understanding complex procedural workflows, and synthesizing information from incident reports to pinpoint underlying systemic failures rather than just superficial errors. By evaluating LLMs’ ability to perform RCA in radiation oncology, this study aims to illuminate their potential utility in clinical workflow optimization and quality improvement, and investigate the current limitations in supporting patient safety efforts. As a proof-of-concept, this exploratory study frames the use of LLMs as an assistive, initial step in the safety review workflow, intended to support human experts rather than replace the comprehensive, multidisciplinary nature of a formal RCA.

## Methods and materials

### RO-ILS study cases

**Fig 1:**
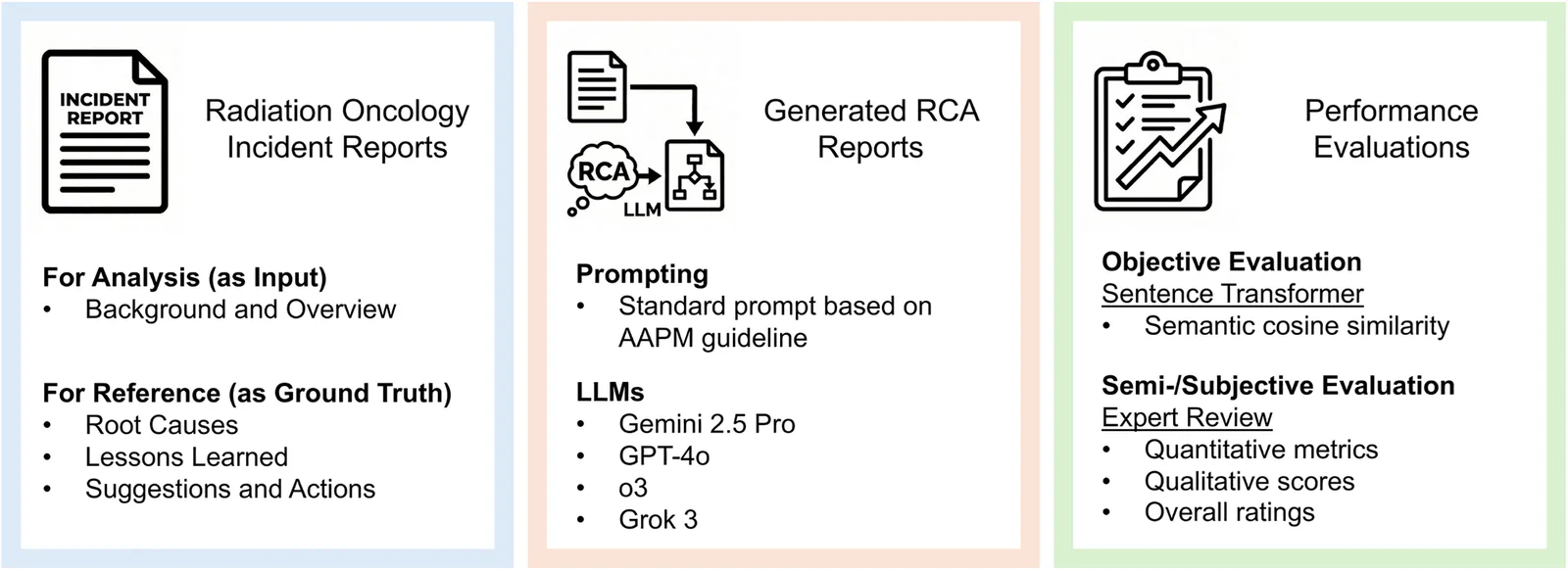
Schematic overview of the study design. (Left) Radiation oncology incident reports from 19 cases were utilized for this study. The “Background and Overview” sections served as inputs for the LLM, while the original sections containing root causes, lessons learned, suggestions, and actions served as the ground truth. (Center) LLMs were utilized to process the event data and generate structured RCA reports, using prompts derived from AAPM guidelines. (Right) Performance evaluation assessed the model outputs using objective, semi-subjective, and subjective metrics for a comprehensive quantification.

Fig 1 outlines the schematic diagram of the study design. We utilized a dataset of nineteen (n = 19) publicly available study cases from the RO-ILS prior to May 2025 [32]. A representative case is provided in File A in S1 Appendix. These cases were selected to represent a variety of incident types and complexities encountered in clinical practice. Each published RO-ILS case typically includes comprehensive documentation comprising:

- Background and Incident Overview: A detailed narrative of the context, sequence of events leading to the incident, and the nature of the incident itself.
- Root Cause(s) and/or Contributing Factors: Expert-identified primary and secondary factors that led to the event.
- Lessons Learned: Key takeaways and insights derived from the analysis of the incident.
- Suggestions and Actions: Recommended corrective and preventative measures to mitigate future occurrences.

The “Background and Incident Overview” section served as the input for each LLM. The remaining sections of the reports, rigorously curated and authored by the Radiation Oncology Healthcare Advisory Council (RO-HAC), a national panel of senior medical physics and patient safety experts from ASTRO, were used as the definitive ground truth reference for evaluation.

### LLM reasoning testing

We tested four state-of-the-art LLMs: Gemini 2.5 Pro (Google) [33], GPT-4o (OpenAI) [34], o3 (OpenAI), and Grok 3 (Grok). For each of the 19 RO-ILS cases, the “Background and Incident Overview” section was provided as input to each LLM. The models were then prompted to perform an RCA and generate associated recommendations using the following standardized prompt in Table 1.

**Table 1:** Standardized prompt used for RCA of RO-ILS cases.

Standardized prompt sentences:
*“Please make a standardized root cause analysis (RCA) for this radiation oncology medical event/incident according to the AAPM standard:* *Simple Framework for RCA includes at least:* • *Chronological sequence– Diagram the flow of events leading up to the incident (including the three “whys”)* • *Cause and Effect Diagramming– Identify the conditions that resulted in the adverse event or close call* • *Causal Statements– Develop root cause and contributing factor statements, actions, and outcome* *After that, please summarize in short bullet points (as many points as needed) for a section of Lessons Learned from this incident, and a section of Suggestions and Actions.”*

Default temperature settings for each respective model (1.0 for Gemini 2.5 Pro, GPT-4o and Grok 3; self-adjusted setting for o3) were used for text generation. The LLM-generated outputs for “Root Cause(s)/Contributing Factors,” “Lessons Learned,” and “Suggestions and Actions” were collected for subsequent evaluation.

### Model evaluation

The performance of each LLM was assessed through a combination of objective, semi-subjective and subjective measures.

### Objective evaluation

We employed text semantic similarity metrics to quantify the overlap between LLM-generated content and the reference text from the corresponding RO-ILS case study. Specifically, cosine similarity was calculated using Sentence Transformers (“all-mpnet-base-v2” model) [35] for the “Root Cause(s)/Contributing Factors,” “Lessons Learned/Suggestions and Actions” sections and the combination of both sections (referred to as the overall texts).

Since some of the 19 raw RO-ILS reports lacked distinct headers, merged sections, or embedded root causes/contributing factors within the “Lessons Learned” or “Suggestions and Actions” text, to conduct a fair and accurate semantic evaluation across this inconsistently formatted raw data, we defined the comparison categories as follows:

- **Root Causes / Contributing Factors:** A direct comparison for cases where the raw RO-ILS report explicitly included this specific section. If the original report lacked this distinct section, the comparison was simply marked as N/A (Cases 1, 5, 6 and 7).
- **Lessons Learned / Suggestions and Actions:** Because the original reports frequently combined these two concepts or omitted one entirely or written with different sub-headers (e.g., “Recommendations” or “Mitigation Strategies”), we combined the LLM’s outputs for both sections and compared them against whatever corresponding text existed in the reference case.
- **Overall Reference Texts:** To have fair comparisons, especially for cases where root causes were not isolated but rather distributed throughout the narrative in different sections, we concatenated all generated sections (“Root Causes / Contributing Factors”, “Lessons Learned”, and “Suggestions and Actions”) into a single corpus and compared it against the full reference text. Because cosine similarity measures broad semantic overlap, this “Overall” category provides a holistic measure of how well the LLM captured the case’s core information, regardless of the specific sub-header it was organized under.

### Semi-subjective evaluation

A panel of five board-certified medical physicists, each with extensive experience in clinical radiation oncology and incident analysis, participated in this phase. Each of the 19 cases, along with the corresponding LLM outputs, was independently evaluated by two physicists to ensure inter-rater reliability. The physicists were blinded to the specific LLM generating each output during their primary review. Following the initial review, scoring discrepancies were discussed and re-evaluated if the difference in objective metrics (precision, recall, F1-score, expert-adjudicated PPV (Positive Predictive Value)) exceeded 0.5, or if the Likert scale scores differed by more than 2. The following aspects were evaluated:

- Root Cause/Contributing Factors Identification: Precision, recall, and F1-score were calculated based on the physicists’ judgment of whether the LLM correctly identified the key root causes and contributing factors present in the ground truth RO-ILS report (from 0 to 1, on numerical values). In addition, expert-adjudicated PPV was calculated based on the physicist’s assessment of the correctness of the identified root causes and contributing factors in the LLM responses (from 0 to 1, on numerical values). Precision, recall, F1-score and expert-adjudicated PPV are defined as follows:
- **Precision**: *Calculated as the number of LLM-generated points that semantically matched any causal elements documented in the expert report, divided by the total number of points generated by the LLM*.
- **Recall**: *Calculated as the number of LLM-generated points that semantically matched any causal elements documented in the expert report, divided by the total number of actual causal elements documented in the ground-truth expert report.*
- **F1-score**: *Calculated based on the following equation*
  $$\begin{array}{c} \text{F1}-\text{score}=\text{2}\times \frac{\text{Precision}\times \text{Recall}}{\text{Precision}+\text{Recall}} \end{array}$$
- **Expert-adjudicated PPV**: *Defined as the number of clinically correct items identified by the LLM, as adjudicated by our expert, divided by the total number of items it generated.* Note that expert-adjudicated PPV in this study was explicitly calculated based on the board-certified physicist’s expert assessment of the correctness of the root causes identified by the LLM. This expert-adjudicated metric captures the true clinical validity when a model identifies an in-depth root cause that happened to be omitted from the non-exhaustive reference text. An item was counted as a match if it captured the core causal factor or logic documented in the corresponding expert report, regardless of exact phrasing.
- Hallucination: Evaluates if the LLM’s response is factually correct and identifies if the LLM’s response includes fabricated, irrelevant, or inaccurate information not supported by the incident description (on binary yes/no: 1 = fabricated information identified, 0 = no fabricated information identified).
- Relevance: Evaluates whether the model’s entire response is pertinent to the specific case study (on a 5-point Likert scale).
- Comprehensiveness: Assesses the comprehensiveness of the identified root causes and actions provided by the LLM (on a 5-point Likert scale).
- Quality of Justification: Assesses the model’s ability to provide clear, logical, and coherent explanations for the identified root causes (on a 5-point Likert scale).
- Quality of Solutions: Assesses the model’s ability to provide reasonable, actionable, and relevant suggestions and actions (on a 5-point Likert scale).

All assessments were evaluated based on the experience and domain knowledge of medical physicists. We referred to these evaluations as semi-subjective.

### Subjective evaluation

Following the detailed semi-subjective assessment, the same physicists were asked to provide an overall subjective score for each LLM’s output per case. This included:

- Reasoning Capability: A rating on a 5-point Likert scale (1 = Very Poor, 5 = Excellent) reflecting the perceived quality of the LLM’s analytical and inferential reasoning in performing the RCA.
- Overall Performance: A rating on a 5-point Likert scale (1 = Very Poor, 5 = Excellent) summarizing the overall utility and quality of the LLM’s complete response for the given case.

The table and detailed rubric used by medical physicists for their evaluations is provided in File B in S1 Appendix.

### Validation with internal cases

As an exploratory qualitative feasibility check, two de-identified internal institutional incident narratives were additionally processed using Gemini 2.5 Pro with the same prompting framework. Both internal reports were strictly locally de-identified to remove any Protected Health Information (PHI) prior to analysis, The evaluation was conducted exclusively using a secure, HIPAA-compliant enterprise API, where data is completely siloed and legally protected from being used for model training.

### Statistical analysis

All quantitative data, including objective metric scores, and subjective Likert scale ratings, were quantified using descriptive statistics across cases for each LLM. A *p*-value of < 0.05 was considered statistically significant. Friedman test was used to assess overall differences among the four models. For metrics demonstrating overall statistical significance (*p* < 0.05), post-hoc pairwise comparisons were conducted using Conover’s test with a Holm-Bonferroni correction to identify specific differences between model pairs.

To assess inter-rater agreement for overlapping paired evaluations, we conducted an inter-rater reliability analysis. For binary variables, we calculated the average exact agreement and Gwet’s AC1 to estimate chance-corrected agreement [36]. For ordinal expert-rated domains assessed on a 5-point Likert scale, we computed both exact and adjacent agreement within ±1 point, along with Gwet’s AC2 for chance correction.

All statistical analyses were performed using custom Python scripts.

## Results

**Fig 2:**
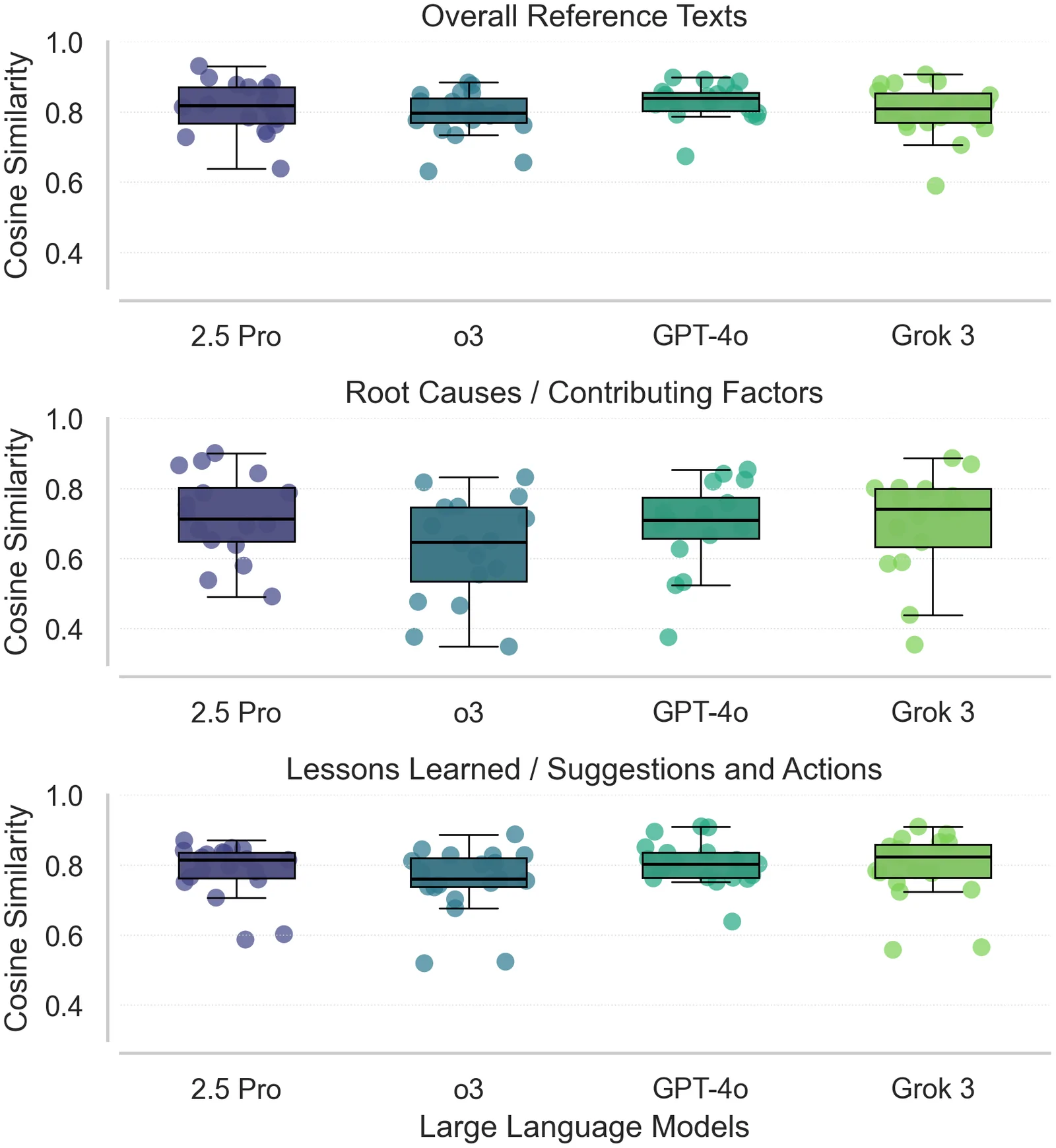
Box plots of cosine similarity scores for LLM outputs compared against three sections of the reference texts. The combined sections (top), “Root Causes / Contributing Factors” (middle), and “Lessons Learned / Suggestions and Actions” (bottom).

RCA reports from the four LLM models were generated following the prompting process. Four representative reports, generated using distinct LLM models, are presented in File C in S1 Appendix. Fig 2 presents box plots of the semantic cosine similarity scores for the four models. For the overall texts, the cosine similarity scores for Gemini 2.5 Pro, o3, GPT-4o and Grok 3 were 0.80 ± 0.08 [mean ± std], 0.79 ± 0.07, 0.83 ± 0.05, and 0.80 ± 0.07, respectively. A similar pattern of performance was observed for the “Contributing Factors / Root Cause” section, and " Lessons Learned / Suggestions and Actions” section. Generally, model GPT-4o demonstrated highest cosine similarity score with the overall reference texts and section “Lessons Learned / Suggestions and Actions,” while Gemini 2.5 Pro achieved highest similarity in the “Root Causes / Contributing Factors” section, as detailed in Table 2.

**Table 2:** Cosine similarity scores of four LLMs for different sections.

Sections	LLM Models				*P*-value
	2.5 Pro	o3	GPT-4o	Grok 3	
Overall Reference Texts	0.80 ± 0.08	0.79 ± 0.07	0.83 ± 0.05	0.80 ± 0.07	0.126
“Root Cause(s) / Contributing Factors”	0.71 ± 0.12 ^a^	0.61 ± 0.15 ^b^	0.68 ± 0.13 ^ab^	0.69 ± 0.14 ^ab^	0.022
“Lessons Learned / Suggestions and Actions”	0.78 ± 0.08 ^a^	0.75 ± 0.09 ^b^	0.80 ± 0.06 ^a^	0.79 ± 0.10 ^a^	0.001

Note: Values represent semantic cosine similarity scores (Mean ± SD). Superscript letters (a, b) denote significant pairwise differences (*p* < 0.05) via Conover’s post-hoc test with Holm-Bonferroni correction; models sharing the same letter are not significantly different The Overall Reference Text scores lack superscripts due to a non-significant Friedman test result (*p* = 0.126).

The Friedman test revealed no statistically significant differences in the overall combined texts among the four models (*p* = 0.126). However, statistically significant differences were observed in specific sub-sections: “Root Causes / Contributing Factors” (*p* = 0.022) and “Lessons Learned / Suggestions and Actions” (*p* = 0.001). For Root Causes/Contributing Factors, post-hoc Conover comparisons showed that Gemini 2.5 Pro had significantly higher cosine similarity than o3 (*p* = 0.038), whereas GPT-4o and Grok 3 were not significantly different from either Gemini 2.5 Pro or o3. For the ‘Lessons Learned / Suggestions and Actions’ section, o3 was significantly outperformed by all other evaluated models (Gemini 2.5 Pro: *p* = 0.032; GPT-4o: *p* = 0.008; Grok 3: *p* = 0.008), while no significant differences were found among Gemini 2.5 Pro, GPT-4o, and Grok 3. Detailed similarity scores for each case study are presented in Fig A in S1 Appendix.

**Fig 3:**
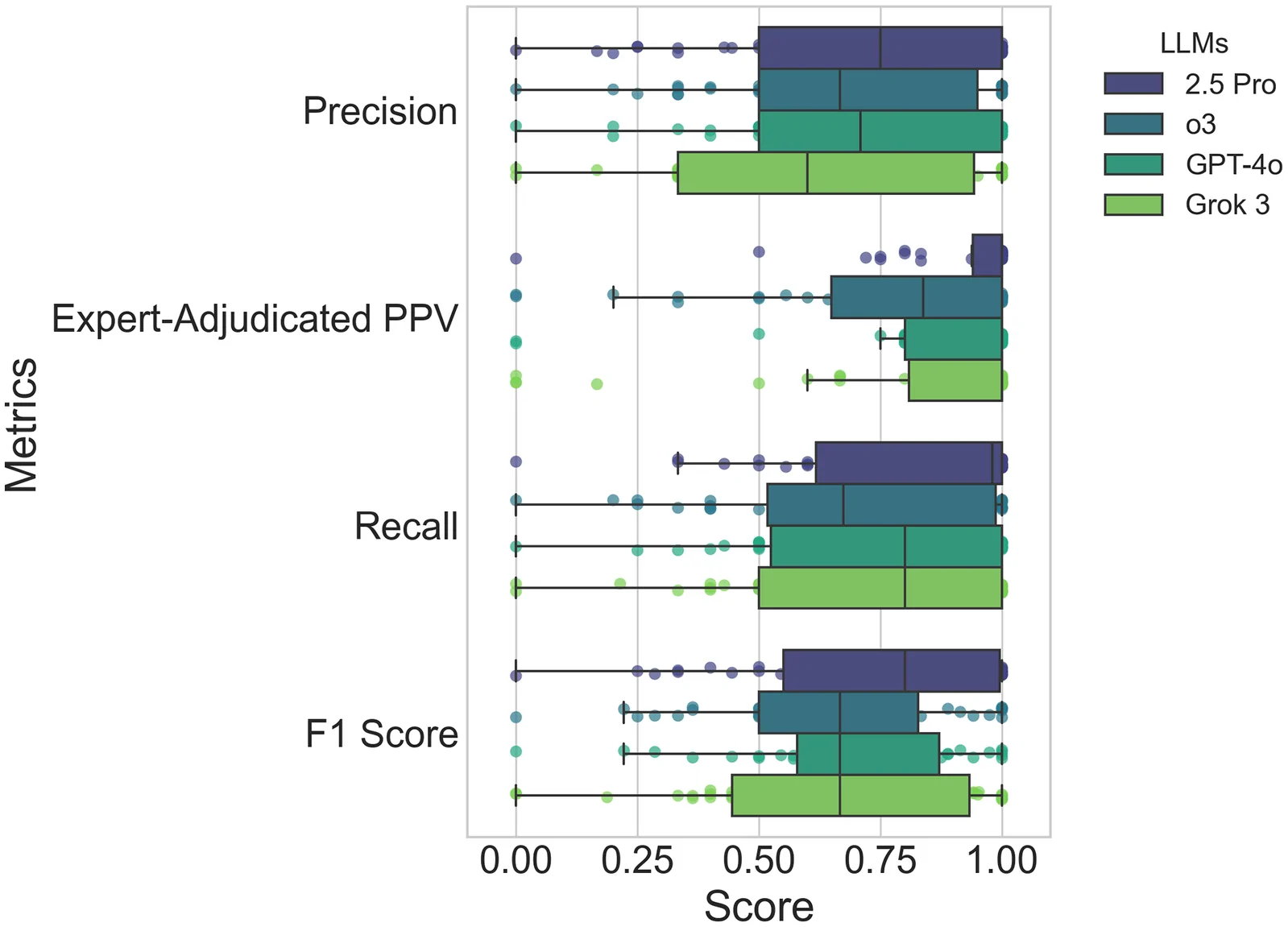
Performance and hallucination analysis for the four evaluated LLMs. Box plot of the distribution of performance metrics including Precision, Expert-Adjudicated PPV, Recall, F1-score.

Table 3 summarizes the performance metrics results from physicists’ evaluation. In the task of identifying root causes and contributing factors, Gemini 2.5 Pro achieved a mean precision of 0.71 ± 0.30, recall of 0.80 ± 0.26, and an F1-score of 0.73 ± 0.27. While aforementioned metrics were based on the root causes documented in the case study report, the expert-adjudicated PPV of root causes identified by Gemini 2.5 Pro was 0.92 ± 0.19 when judged by the expert physicist panel. LLMs of o3, GPT-4o and Grok 3 displayed a similar trend (Fig 3 and Table 3).

**Table 3:** Performance metrics and hallucination evaluation for the four LLMs.

Performance Metrics	Model				*P*-value
	2.5 Pro	o3	GPT-4o	Grok 3	
Precision	0.70 ± 0.30	0.66 ± 0.27	0.70 ± 0.28	0.61 ± 0.31	0.091
Expert-adjudicated PPV	0.92 ± 0.19 ^a^	0.77 ± 0.29 ^b^	0.88 ± 0.24 ^a^	0.82 ± 0.3 ^ab^	0.002
Recall	0.80 ± 0.26	0.69 ± 0.27	0.75 ± 0.26	0.72 ± 0.30	0.113
F1 Score	0.73 ± 0.27	0.65 ± 0.25	0.69 ± 0.23	0.64 ± 0.29	0.225
Hallucination	0.11 ± 0.31 ^a^	0.61 ± 0.50 ^b^	0.32 ± 0.47 ^a^	0.29 ± 0.46 ^a^	< 0.001

Note: Hallucination was scored as a binary metric (0 = Absent, 1 = Present). Precision, Recall, F1-score, and Expert-adjudicated PPV range from 0 to 1. Values are presented as Mean ± Standard Deviation or percentages. Superscript letters (a, b) denote statistical significance (*p* < 0.05) based on Conover’s post-hoc test with Holm-Bonferroni correction. No superscripts are assigned to Precision, Recall, and F1-score as their overall Friedman test yielded *p* ≥ 0.05.

No statistically significant differences among the four models in precision (*p* = 0.091), recall (*p* = 0.113), and F1-score (*p* = 0.225), indicating that all evaluated LLMs possess comparable baseline capabilities in extracting relevant causal items (Table 3). However, statistical analysis revealed a significant difference in expert-adjudicated PPV across the evaluated models (*p* = 0.002). Both Gemini 2.5 Pro and GPT-4o significantly outperformed the o3 model (*p* = 0.011 for both comparisons), while no significant difference was found between Gemini 2.5 Pro and GPT-4o (*p* = 0.945).

Instances of hallucination, defined as fabricated or inaccurate information, were observed in 11% of cases for Gemini 2.5 Pro, 61% for o3, and 32% for GPT-4o, and 29% for Grok 3 respectively on average (Table 3). The hallucination score differed significantly among the models (*p* < 0.001). Gemini 2.5 Pro, GPT-4o and Grok 3 exhibited significantly lower hallucination rates compared to o3 (*p* < 0.001, *p* = 0.047 and *p* = 0.047, respectively), while no significant differences were observed among Gemini 2.5 Pro, GPT-4o, and Grok 3.

**Fig 4:**
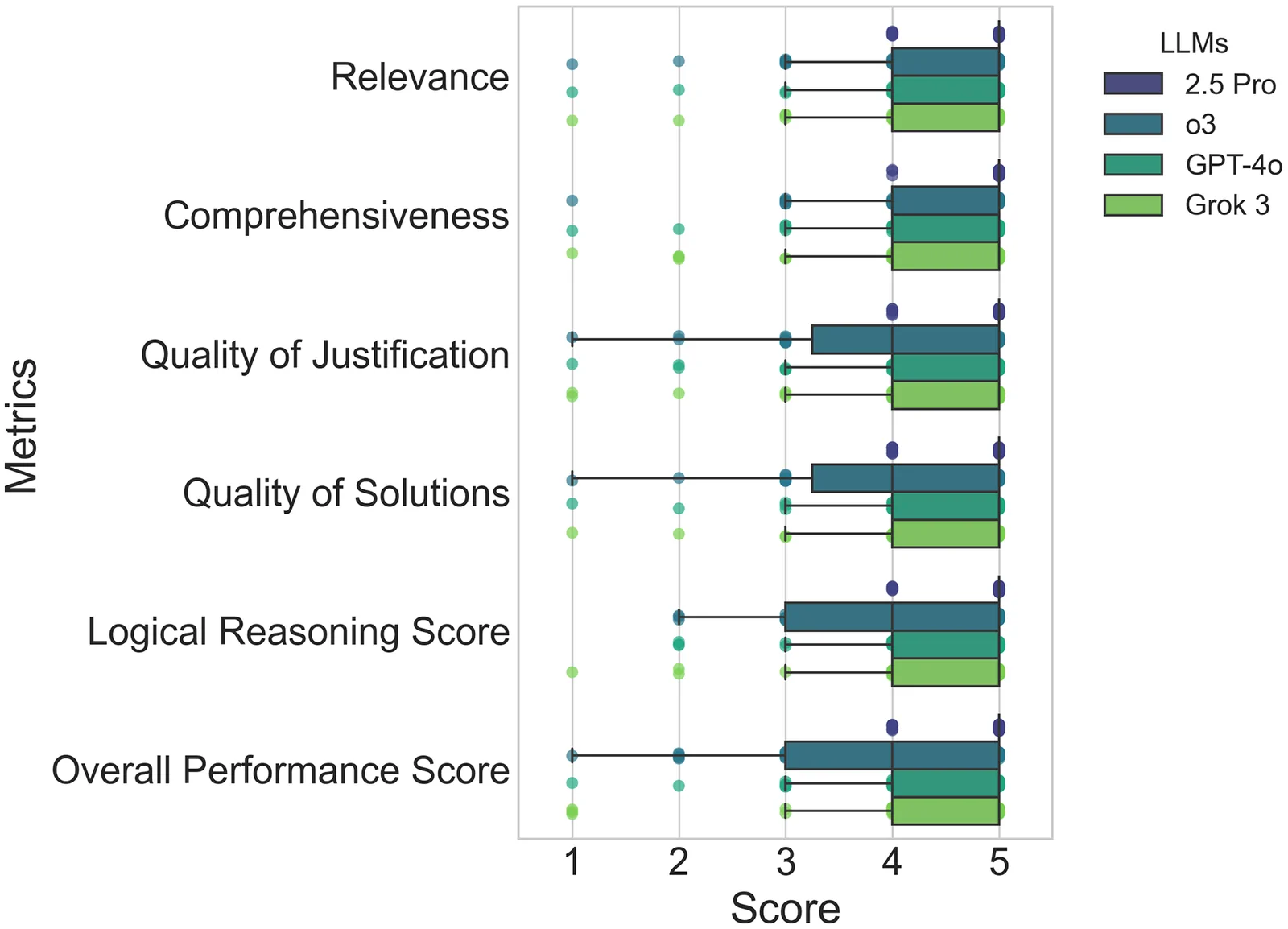
Evaluation of performance for four LLMs. Box plot of the score distribution for six key metrics: Relevance, Comprehensiveness, Quality of Justification, Quality of Solutions, Logical Reasoning, and Overall Performance.

All models demonstrated satisfactory performance across the evaluated criteria, including Relevance, Comprehensiveness, Quality of Justification, Quality of Solutions, as well as in the subjective assessments of Logical Reasoning and Overall Performance (Fig 4 and Table 4). All performance criteria and overall ratings were found to be statistically significantly different among models (*p* < 0.001). Among the four models, Gemini 2.5 Pro generally received the highest expert-rated scores, although the pattern of significant pairwise differences varied by domain. GPT-4o and/or Grok 3 showed statistically overlapping performance with Gemini 2.5 Pro in several metrics. Specifically, Gemini 2.5 Pro significantly outperformed all other models in Quality of Justification (o3: *p* < 0.001; GPT-4o: *p* = 0.011; Grok 3: *p* = 0.023) and the Overall Performance Score (o3: *p* < 0.001; GPT-4o: *p* = 0.026; Grok 3: *p* = 0.026).

**Table 4:** Model performances in Relevance, Comprehensiveness, Quality of Justification, Quality of Solutions and subjective ratings of Logical Reasoning Score and Overall Performance Score of four LLMs.

Performance Metrics	Model				*P*-value
	2.5 Pro	o3	GPT-4o	Grok 3	
Relevance	4.79 ± 0.41 ^a^	4.21 ± 1.02 ^b^	4.45 ± 0.92 ^ab^	4.50 ± 0.95 ^ab^	< 0.001
Comprehensiveness	4.92 ± 0.27 ^a^	4.11 ± 0.92 ^b^	4.42 ± 0.98 ^ab^	4.39 ± 1.08 ^b^	< 0.001
Quality of Justification	4.84 ± 0.37 ^a^	4.11 ± 1.06 ^b^	4.32 ± 1.02 ^b^	4.34 ± 1.10 ^b^	< 0.001
Quality of Solutions	4.79 ± 0.41 ^a^	4.08 ± 1.00 ^b^	4.42 ± 0.95 ^ab^	4.50 ± 0.92 ^a^	< 0.001
Logical Reasoning Score	4.79 ± 0.41 ^a^	4.08 ± 1.00 ^c^	4.37 ± 0.91 ^bc^	4.42 ± 0.98 ^ab^	< 0.001
Overall Performance Score	4.82 ± 0.39 ^a^	3.76 ± 1.17 ^c^	4.26 ± 0.98 ^b^	4.26 ± 1.13 ^b^	< 0.001

Note: Subjective scores were evaluated on a 5-point Likert scale (1 = Very Poor, 5 = Excellent). Values are presented as Mean ± Standard Deviation. Different superscript letters (a, b, c) within a row denote statistically significant differences (*p* < 0.05) based on Conover’s post-hoc test with Holm-Bonferroni correction. Models sharing the same superscript letter are not significantly different.

Inter-rater agreement metrics are summarized in Table 5. For binary hallucination scoring, exact agreement was 78.9%, and Gwet’s AC1 was 0.623. For the ordinal domains, average exact agreement ranged from 43.4% to 51.3%, whereas adjacent agreement (±1 point tolerance) ranged from 76.3% to 82.9%, indicating that large rating discrepancies were relatively uncommon. Gwet’s AC2 ranged from 0.590 to 0.707. Overall, these findings suggest moderate to high agreement, while also underscoring the subjective and context-dependent nature of RCA evaluation.

**Table 5:** Inter-rater agreement metrics across evaluated rating domains.

Evaluation Metric	Average Exact Agreement (%)	Average Adjacent Agreement (±1 point) (%)	Gwet’s AC1 / AC2
Hallucination	78.9	N/A	0.623
Relevance	47.4	82.9	0.707
Comprehensiveness	51.3	76.3	0.677
Quality of Justification	44.7	76.3	0.606
Quality of Solutions	46.1	78.9	0.677
Logical Reasoning Score	46.1	81.6	0.682
Overall Performance Score	43.4	76.3	0.590

Note: Hallucination was scored as a binary variable (0/1); therefore, adjacent agreement (±1 point) is not applicable for this domain, and Gwet’s AC1 is reported. For ordinal expert-rated domains, adjacent agreement was defined as paired ratings within ±1 point on the 5-point Likert scale, and Gwet’s AC2 is reported as the chance-corrected agreement coefficient.

As a qualitative illustration, the same prompting framework was applied to two de-identified internal incident narratives, with example outputs provided in File D in S1 Appendix. The framework demonstrated promising performance in both internal verification cases.

## Discussion

Our study demonstrates the promising, albeit still developing, reasoning capabilities of leading LLMs when applied to the complex task of RCA for radiation oncology incidents. The findings indicate that LLM models can generate RCAs that exhibit considerable alignment with expert-derived analyses. Specifically, the LLMs achieved an average semantic similarity score as high as 0.83 when comparing their generated root causes, lessons learned, and suggested actions against the gold-standard reference texts. Additionally, the evaluated LLMs achieved favorable expert-rated performance overall, with some differences across models in hallucination rates and subjective quality ratings. This suggests an emerging potential for LLMs to assist in the initial stages of incident analysis, potentially streamlining the process for human experts.

Despite their advanced capabilities, we observed persistent presence of hallucinations across all evaluated LLMs in this task. Although the 11% hallucination rate observed in the top-performing model is not negligible, it may be tolerable in non-patient-facing applications, and only with mandatory human review. In our qualitative observations, the hallucinations encountered were generally minor, typically presenting as unsupported explanatory details that were not sufficiently justified by the source narrative, rather than clinically misleading statements. They usually did not materially alter the suggested corrective actions. In this context, LLMs can assist by generating initial drafts or highlighting potential contributing factors that might be overlooked by human reviewers. However, we cannot exclude the possibility of severe hallucinations in broader practice, where they could potentially impact clinical or operational decisions. Given such risks, we emphasize a mandatory human-in-the-loop workflow. The LLM serves merely as a first-pass assistive tool for structural organization. All extracted root causes and suggested actions must be systematically verified by board-certified physicists and radiation oncologists before conclusions are drawn or clinical decisions are affected. In the current study, we did not formally quantify or categorize hallucination severity. This will be the focus of more comprehensive future research.

Interestingly, we observed a case in which one of the four LLMs failed to produce an output for a root cause analysis, despite being strongly prompted to do so (File E in S1 Appendix). Upon expert review, it was confirmed that the incident did not involve any patient harm or workflow failure and merely represented a routine alert during daily image-guided treatment. Although the model’s response failed all predefined evaluation metrics, it ultimately offered the true interpretation of the scenario. In contrast, the other three models generated RCA-style explanations that were not adequately supported by the incident context. This observation suggests that the model may, in some cases, correctly identify when a narrative does not support a meaningful RCA interpretation.

LLMs have shown expert level in tasks related to medical question answering [37,38] and reasoning [39]. They have also been increasingly integrated into radiation oncology [40] for tasks such as language-assisted contouring [41,42] and treatment planning [43,44], clinical information summarization and retrieval [45], clinical question and inquiry response [46,47], and education [48]. It has also been utilized to categorize radiation oncology incidents and triage critical events for immediate intervention [49,50]. These valuable applications primarily capitalize on the text-processing and understanding strengths inherent to LLMs. Furthermore, the integration of AI and LLMs aligns with broader interdisciplinary trends in precision medicine. These range from advanced cardiovascular imaging diagnostics [51] to mechanistic studies addressing radioresistance (e.g., microRNA targeting) [52] and the development of novel nanoparticle radiosensitizers to enhance therapeutic efficacy [53].

In contrast, our research advances the field by utilizing the generative capabilities of frontier LLMs to translate unstructured incident narratives into a standardized RCA framework, moving beyond simple event classification toward structured causal reasoning. This is particularly critical in radiation oncology, where incident narratives are dense with medical physics jargon and errors frequently cascade across a highly interconnected workflow, procedures, equipment, and multidisciplinary handoffs.

The task of performing RCA in radiation oncology incidents also presents distinct challenges compared to general reasoning tasks often evaluated by standard LLM benchmarks. While general benchmarks test logical deduction, common sense, or factual recall, RCA in this domain requires a deep understanding of highly specific medical and technical knowledge, intricate clinical workflows, and human factors unique to radiation therapy. The incidents are often complex, multi-causal, and require not just logical deduction but also abductive reasoning to infer the most plausible underlying causes from incomplete narratives. Furthermore, unlike many well-defined reasoning tasks, RCA in this context is an inherently open-ended problem. While structured by frameworks like the AAPM recommendations, the identification of specific causes and the formulation of effective “Suggestions and Actions” demand a degree of contextual understanding and real-world problem-solving that goes beyond simple pattern matching. The ability of the tested LLMs to produce relevant outputs in this specialized, logic-driven yet open-ended task suggests an encouraging capacity for detailed, domain-aware reasoning of LLMs.

Evaluating such sophisticated reasoning capabilities accurately is an ongoing challenge. In this study, we employed a dual approach, combining objective text similarity metrics (Sentence Transformer) with comprehensive semi-subjective and subjective evaluations by experienced medical physicists. While objective metrics offer quantifiable measures of textual overlap with ground truth reports, they may not fully capture the semantic correctness, the subtlety of identified causal links, or the practical utility of the generated suggestions. The qualitative assessments by domain experts, focusing on aspects like hallucination, relevance, and the quality of justifications and solutions, provided crucial insights into these aspects of performance. However, these expert ratings also retained an inherent degree of subjectivity. Although exact inter-rater agreement was modest in several domains, adjacent agreement was relatively high, suggesting that large rating discrepancies were uncommon. Gwet’s AC1/AC2 provided chance-corrected agreement estimates that were more appropriate for the binary hallucination and ordinal expert-rated domains, particularly given the restricted score range and skewed distribution of Likert ratings [36]. Nevertheless, these agreement results should still be interpreted in light of the inherent subjectivity and context-dependent nature of RCA judgments. The natural variability in expert ratings highlights that causal interpretation often depends on an individual’s unique clinical experience. This subjectivity reinforces that LLM-generated reports should be utilized as a collaborative starting point for multidisciplinary discussions rather than definitive conclusions. The robust quantification of “reasoning” in LLMs, especially for domain-specific, high-stakes tasks, is still an evolving area of research. Further investigation is imperative to develop and validate more sophisticated and reliable metrics that can truly capture the depth and accuracy of LLM-driven analytical processes.

The current investigation utilized publicly available RO-ILS cases, which provided a standardized and transparent dataset for this initial evaluation. However, the full potential of LLMs in this context lies in their secure, local deployment within healthcare institutions. Because real-world incident reports contain highly sensitive Protected Health Information (PHI), utilizing public, web-based LLM interfaces poses significant ethical and legal risks. Future clinical implementation must rely on HIPAA-compliant enterprise-level APIs, or locally hosted open-source models (e.g., Gemma 4) operating strictly within a hospital’s private network to ensure data security. Integrating LLMs with an institution’s internal incident reporting system could enable the analysis of a much larger, more diverse, and highly contextualized dataset of local incidents. Such deployment would also require institution-specific governance and oversight structures to define workflow integration points, review responsibility, and accountability for downstream quality-management decisions. Such an approach could offer powerful insights into institution-specific vulnerabilities, help optimize local clinical workflows, and identify systemic weaknesses in the chain of clinical practice. This local application could transform RCA from a primarily reactive process to a more proactive and continuous quality improvement mechanism. The development of agentic LLM workflows represents the next frontier, where a multi-agent system embedded in the clinical environment could autonomously surveil and triage events, generate initial root cause analyses, and coordinate communication with relevant personnel and safety committees, ultimately establishing a responsive, automated framework dedicated to improving patient safety.

The narrative descriptions in the initial “Background and Incident Overview” as model inputs are often brief and inherently limited. However, this is precisely the real-world clinical scenario we aimed to simulate. Initial incident reports filed by clinical staff are frequently incomplete. In our analysis, we observed a weak positive correlation between the length of an incident narrative, as measured by word count, and the performance metrics of our model, including recall, precision, F1-score, and expert-adjudicated PPV (Fig B in S1 Appendix). Although the correlation is not strong, it suggests that more detailed descriptions can contribute to better model performances. This finding underscores the critical importance of submitting incident reports with narratives that are both accurate and comprehensive. Our observation aligns with the principles outlined by the AAPM’s Task Group 288 (TG-288), which provides formal guidance for composing effective radiotherapy event narratives [54]. Adhering to guidance recommendations suggested by TG-288 can help ensure that incident learning systems receive high-quality data, thereby enhancing the potential use of LLMs for identifying vulnerabilities and improving patient safety.

Another structured safety methodology advocated by the AAPM is the TG-100, which specifically promotes Failure Mode and Effects Analysis (FMEA) as a proactive risk assessment tool in radiation therapy [55]. FMEA systematically identifies potential failure modes in treatment processes, assesses their risk based on severity, occurrence, and detectability, and prioritizes actions to mitigate these risks before incidents occur. While FMEA is highly effective, it is resource-intensive and heavily reliant on expert judgment. Our current research suggests that these LLM models could potentially support and enhance FMEA processes. Furthermore, future integration of LLM-driven approaches could assist in objectively quantifying risk factors, providing standardized, data-driven assessments that complement expert analysis, thereby potentially reducing human bias and variability.

The performance of LLMs is heavily dependent on prompt engineering. To determine the optimal strategy for eliciting deep clinical reasoning, prior to executing our comprehensive multi-model evaluation, we conducted a preliminary prompt tuning phase. To demonstrate, we showed the output from a “naive” prompt (“*Please make a standardized root cause analysis (RCA) for this radiation oncology medical event/incident*”) on a representative case (File F in S1 Appendix). Compared to the prompt using AAPM standardized RCA guidelines as in the current study, while the “naive” prompt successfully identified broad systemic failures, the AAPM-guided prompt uncovered highly granular, domain-specific workflow deficits, such as precise training gaps regarding small bowel migration, and accurately distinguished primary root causes from enabling contributing factors. This underscores the importance of structured, domain-specific prompt engineering. By explicitly instructing the model to establish a chronological sequence, iteratively ask “whys,” map cause-and-effect conditions, and subsequently deduce formal causal statements, this prompt inherently served as a domain-specific Chain of Thought (CoT) mechanism, elevating overall model performance.

Looking forward, there is considerable scope for enhancing the evaluation methodologies for LLM-generated RCAs. Beyond current text similarity and expert rating systems, future studies could explore the application of more advanced quantification techniques, such as those derived from causal inference frameworks. This might involve assessing the LLMs’ ability to not only identify contributing factors but also to correctly map the causal pathways and interdependencies between them, perhaps even by evaluating their capacity to construct or critique causal diagrams based on incident narratives. Such methods could provide a more rigorous assessment of the depth of an LLM’s understanding of the complex cause-and-effect relationships inherent in radiation oncology incidents, moving beyond correlational analysis to a more profound evaluation of genuine causal reasoning. Continued research in these directions will be vital for responsibly harnessing the power of LLMs to augment human expertise in the critical domain of patient safety.

Because the incident narratives may contain implicit causal clues, the task performed by the LLMs may reflect high-level structured summarization rather than independent, *de novo* reasoning. While the evaluated models have demonstrated high alignment with expert ratings, this performance may also partially reflect advanced semantic summarization and sophisticated pattern matching with established RCA frameworks, either from input descriptions or training data, rather than fully independent mechanistic reasoning. Genuine root cause analysis remains a multifaceted process requiring physical workflow investigation, system log reviews, and multidisciplinary deliberation, actions an LLM inherently cannot perform. Therefore, it is critical to distinguish sophisticated pattern alignment from true mechanistic reasoning, reinforcing the conclusion that human-led RCA is an irreplaceable necessity.

By grounding our prompt engineering in AAPM guidelines, this study validates the feasibility of using LLMs for RCA. The translational goal of this application is to convert unstructured RO-ILS narratives into highly organized, actionable insights. By prompting LLMs to generate standardized categorical tags rather than free-text responses, we can produce datasets that directly support continuous monitoring, quantitative trend analysis, and systematic workflow optimization. To demonstrate this practical utility, our subsequent investigation successfully applied this LLM-driven RCA framework to 254 internal institutional RO-ILS events, systematically populating a structured, queryable incident database [56].

A potential limitation of this study is the reliance on publicly available radiation oncology incident reports, which introduces the risk that these texts were included in the LLMs’ training datasets (data contamination). However, the distinct variations in performance observed across the different models suggest that they are generating responses based on inference rather than merely reproducing memorized training data. To mitigate this concern, we validated the approach using two internal institutional cases. These private cases yielded satisfactory performance consistent with the public dataset, supporting the generalizability of our findings. Because no expert-verified reference analyses were available for these internal cases, they are presented as illustrative feasibility examples for proof-of-concept rather than as part of the formal quantitative benchmark. Nevertheless, future evaluation on larger datasets of unseen institutional reports with expert-annotated reference analyses will be necessary to more rigorously assess real-world generalizability.

The reliance on public case study reports also constrains the diversity and volume of the analyzed cases. Consequently, the current dataset may not fully capture the breadth of clinical scenarios encountered across the full spectrum of radiation oncology, particularly regarding specialized modalities such as low-dose-rate (LDR) brachytherapy, adaptive radiation therapy, and proton therapy. This limitation could affect the generalizability of our findings when applied to the distinct workflows associated with these complex or less common scenarios. Therefore, the current study should be considered a proof-of-concept pilot investigation. In addition, the limited sample size, coupled with the inherent complexity of radiotherapy modalities, precluded an analysis of case-type sensitivity in this study. While this cohort size was adequately powered to identify statistically significant differences in models exhibiting large effect sizes, we recognize that a larger sample is necessary to detect marginal performance differences.

Furthermore, these publicly available cases are curated and selected educational reports that are often cleaner than raw clinical narratives. This curation removes the “noise,” ambiguity, and incomplete information typical of raw clinical incident reports. Consequently, the performance observed on this dataset likely represents a best-case scenario. While the successful evaluation of our two internal institutional cases demonstrated highly satisfactory performance on real-world data, we will incorporate a much larger cohort of raw institutional RO-ILS reports in future studies. This expansion will ensure a more comprehensive evaluation of LLM performance in real-world clinical settings, spanning varied clinical contexts and treatment techniques.

To ensure a fair, “out-of-the-box” comparison and preserve the advanced reasoning capabilities required for root cause analysis, all models in the current study were evaluated using their default temperature settings. Consequently, the inherent stochasticity of these models remains a limitation, as outputs may vary between generations. Future investigations must incorporate rigorous, multi-run reproducibility testing to fully characterize and quantify this variability.

A notable limitation of this study is the rapid, continuous evolution of LLMs, as the systems evaluated reflect the frontier capabilities available strictly during the study period. As newer iterations continue to emerge, baseline performance metrics, reasoning depth, and overall clinical accuracy will inevitably shift. However, the rigorous, dual-evaluation framework established herein is inherently model-agnostic, providing a standardized methodology to benchmark the clinical efficacy of future AI systems for RCA in radiation oncology.

## Conclusions

This study demonstrates the emerging potential of using LLMs for RCA in radiation oncology incidents. By prompting four state-of-the-art LLMs to analyze RO-ILS incident reports, we observed that these models can generate RCA outputs with high semantic similarity to expert-generated references and receive favorable evaluations from experienced medical physicists. While important limitations such as hallucinations remain, our proof-of-concept study suggests its promising use in clinical applications such as workflow optimization, quality improvement and incident analysis under mandatory human supervision in support of patient safety.

## Supporting information

S1 Appendix Fig A. Cosine similarity scores for LLM outputs compared against three sections of the reference texts. **Fig B.** Scatter plot of prompt text length vs model performance. **File A.** A representative RO-ILS case report used in this study. **File B.** Evaluation table and rubric used by medical physicists. **File C.** Representative output samples generated by the four evaluated LLMs. **File D** Two internal institutional cases processed by Gemini 2.5 Pro. **File E.** Failed-generation / “unable to perform RCA” example. **File F.** Comparative ablation study on prompt sensitivity.
(PDF)

## References

[1] Teo ZL, Thirunavukarasu AJ, Elangovan K, Cheng H, Moova P, Soetikno B, et al. Generative artificial intelligence in medicine. Nat Med. 2025;31(10):3270–82. doi: 10.1038/s41591-025-03983-2 41053447

[2] Meskó B, Topol EJ. The imperative for regulatory oversight of large language models (or generative AI) in healthcare. NPJ Digit Med. 2023;6(1):120. doi: 10.1038/s41746-023-00873-0 37414860 PMC10326069

[3] Hao Y, Qiu Z, Holmes J, Löckenhoff CE, Liu W, Ghassemi M, et al. Large language model integrations in cancer decision-making: a systematic review and meta-analysis. NPJ Digit Med. 2025;8(1):450. doi: 10.1038/s41746-025-01824-7 40676129 PMC12271406

[4] Plaat A, Wong A, Verberne S, Broekens J, van Stein N, Back T. Reasoning with large language models, a survey. 2024. doi: arXiv:240711511

[5] Huang J, Chang KC-C. Towards reasoning in large language models: A survey. arXiv preprint. 2022. doi: 10.48550/arXiv.221210403

[6] Li ZZ, Zhang D, Zhang ML, Zhang J, Liu Z, Yao Y. From system 1 to system 2: A survey of reasoning large language models. arXiv preprint. 2025. doi: 10.48550/arXiv.250217419 41289126

[7] Fedus W, Zoph B, Shazeer N. Switch transformers: Scaling to trillion parameter models with simple and efficient sparsity. Journal of Machine Learning Research. 2022;23(120):1–39.

[8] Brown T, Mann B, Ryder N, Subbiah M, Kaplan JD, Dhariwal P, et al. Language models are few-shot learners. Advances in neural information processing systems. 2020;33:1877–901.

[9] Cai W, Jiang J, Wang F, Tang J, Kim S, Huang J. A Survey on Mixture of Experts in Large Language Models. IEEE Trans Knowl Data Eng. 2025;:1–20. doi: 10.1109/tkde.2025.3554028

[10] Patil A, Jadon A. Advancing reasoning in large language models: promising methods and approaches. 2025. doi: arXiv:250203671

[11] Gunel B, Du J, Conneau A, Stoyanov V. Supervised contrastive learning for pre-trained language model fine-tuning. arXiv preprint. 2020. doi: 10.48550/arXiv.2011.01403

[12] Bai Y, Jones A, Ndousse K, Askell A, Chen A, DasSarma N. Training a helpful and harmless assistant with reinforcement learning from human feedback. arXiv preprint. 2022. doi: 10.48550/arXiv.2204.05862

[13] Xu F, Hao Q, Zong Z, Wang J, Zhang Y, Wang J. Towards large reasoning models: A survey of reinforced reasoning with large language models. 2025. doi: arXiv:250109686 10.1016/j.patter.2025.101370 PMC12546433 41142903

[14] Wei J, Wang X, Schuurmans D, Bosma M, Ichter B, Xia F, et al. Chain-Of-Thought Prompting Elicits Reasoning in Large Language Models. In: Advances in Neural Information Processing Systems 35, 2022. 24824–37. 10.52202/068431-1800

[15] Yao S, Yu D, Zhao J, Shafran I, Griffiths T, Cao Y, et al. Tree of Thoughts: Deliberate Problem Solving with Large Language Models. In: Advances in Neural Information Processing Systems 36, 2023. 11809–22. 10.52202/075280-0517

[16] Kojima T, Gu S (Shane), Reid M, Matsuo Y, Iwasawa Y. Large Language Models Are Zero-Shot Reasoners. In: Advances in Neural Information Processing Systems 35, 2022. 22199–213. 10.52202/068431-1613

[17] Srivastava A, Rastogi A, Rao A, Shoeb AA, Abid A, Fisch A. Beyond the imitation game: quantifying and extrapolating the capabilities of language models. Transactions on Machine Learning Research. 2023.

[18] Chen M, Tworek J, Jun H, Yuan Q, Pinto HPDO, Kaplan J. Evaluating large language models trained on code. 2021. doi: 10.48550/arXiv.2107.03374

[19] Hendrycks D, Burns C, Basart S, Zou A, Mazeika M, Song D. Measuring massive multitask language understanding. In: 2020. https://arxiv.org/abs/2009.03300

[20] Cobbe K, Kosaraju V, Bavarian M, Chen M, Jun H, Kaiser L, et al. Training verifiers to solve math word problems. In: 2021. https://arxiv.org/abs/2110.14168

[21] Rein D, Hou BL, Stickland AC, Petty J, Pang RY, Dirani J. Gpqa: A graduate-level google-proof q&a benchmark. In: 2024.

[22] Huang Y, Yang LF. Gemini 2.5 Pro capable of winning gold at IMO 2025. In: 2025. https://doi.org/arXiv:250715855

[23] Ferrag MA, Tihanyi N, Debbah M. From llm reasoning to autonomous ai agents: A comprehensive review. 2025. https://arxiv.org/abs/250419678

[24] Huang L, Yu W, Ma W, Zhong W, Feng Z, Wang H, et al. A Survey on Hallucination in Large Language Models: Principles, Taxonomy, Challenges, and Open Questions. ACM Trans Inf Syst. 2025;43(2):1–55. doi: 10.1145/3703155

[25] Holmberg O, McClean B. Preventing treatment errors in radiotherapy by identifying and evaluating near misses and actual incidents. J Radiother Pract. 2002;3(1):13–25. doi: 10.1017/s1460396902000122

[26] Wachter RM. Understanding patient safety: McGraw-Hill Education; 2008.

[27] Ford EC, Evans SB. Incident learning in radiation oncology: A review. Med Phys. 2018;45(5):e100–19. doi: 10.1002/mp.12800 29419944

[28] Evans SB, Viscariello NN. Learning from ten-years of RO-Ils data: how to avoid common error pathways and engage the entire team. In: 2025.

[29] Hoopes DJ, Dicker AP, Eads NL, Ezzell GA, Fraass BA, Kwiatkowski TM, et al. RO-ILS: Radiation Oncology Incident Learning System: A report from the first year of experience. Pract Radiat Oncol. 2015;5(5):312–8. doi: 10.1016/j.prro.2015.06.009 26362705

[30] Williams PM. Techniques for root cause analysis. Baylor University Medical Center Proceedings. Taylor & Francis. 2001.

[31] Latino RJ. Patient safety: the PROACT root cause analysis approach. CRC Press. 2008.

[32] RO-ILS Education. https://www.astro.org/practice-support/quality-and-safety/ro-ils/ro-ils-education

[33] Team G, Anil R, Borgeaud S, Alayrac JB, Yu J, Soricut R. Gemini: a family of highly capable multimodal models. 2023. https://arxiv.org/abs/2312.11805

[34] Achiam J, Adler S, Agarwal S, Ahmad L, Akkaya I, Aleman FL, et al. Gpt-4 technical report. 2023. https://arxiv.org/abs/2303.08774

[35] Song K, Tan X, Qin T, Lu J, Liu TY. Mpnet: Masked and permuted pre-training for language understanding. Advances in Neural Information Processing Systems. 2020;33:16857–67.

[36] Gwet KL. Computing inter-rater reliability and its variance in the presence of high agreement. Br J Math Stat Psychol. 2008;61(Pt 1):29–48. doi: 10.1348/000711006X126600 18482474

[37] Singhal K, Azizi S, Tu T, Mahdavi SS, Wei J, Chung HW, et al. Large language models encode clinical knowledge. Nature. 2023;620(7972):172–80. doi: 10.1038/s41586-023-06291-2 37438534 PMC10396962

[38] Singhal K, Tu T, Gottweis J, Sayres R, Wulczyn E, Amin M, et al. Toward expert-level medical question answering with large language models. Nat Med. 2025;31(3):943–50. doi: 10.1038/s41591-024-03423-7 39779926 PMC11922739

[39] Lucas MM, Yang J, Pomeroy JK, Yang CC. Reasoning with large language models for medical question answering. J Am Med Inform Assoc. 2024;31(9):1964–75. doi: 10.1093/jamia/ocae131 38960731 PMC11339506

[40] Liu Z, Li Y, Shu P, Zhong A, Jiang H, Pan Y, et al. Radiology-GPT: A large language model for radiology. Meta-Radiology. 2025;3(2):100153. doi: 10.1016/j.metrad.2025.100153

[41] Rajendran P, Chen Y, Qiu L, Niedermayr T, Liu W, Buyyounouski M, et al. Autodelineation of Treatment Target Volume for Radiation Therapy Using Large Language Model-Aided Multimodal Learning. Int J Radiat Oncol Biol Phys. 2025;121(1):230–40. doi: 10.1016/j.ijrobp.2024.07.2149 39117164 PMC13041240

[42] Rajendran P, Yang Y, Niedermayr TR, Gensheimer M, Beadle B, Le Q-T, et al. Large language model-augmented learning for auto-delineation of treatment targets in head-and-neck cancer radiotherapy. Radiother Oncol. 2025;205:110740. doi: 10.1016/j.radonc.2025.110740 39855601 PMC11956750

[43] Dong Z, Chen Y, Gay H, Hao Y, Hugo GD, Samson P, et al. Large-language-model empowered 3D dose prediction for intensity-modulated radiotherapy. Med Phys. 2025;52(1):619–32. doi: 10.1002/mp.17416 39316523

[44] Wei S, Hu A, Liang Y, Yang J, Yu L, Li W, et al. Feasibility study of automatic radiotherapy treatment planning for cervical cancer using a large language model. Radiat Oncol. 2025;20(1):77. doi: 10.1186/s13014-025-02660-5 40375332 PMC12083153

[45] Wang P, Liu Z, Li Y, Holmes J, Shu P, Zhang L, et al. Fine-tuning open-source large language models to improve their performance on radiation oncology tasks: A feasibility study to investigate their potential clinical applications in radiation oncology. Med Phys. 2025;52(7):e17985. doi: 10.1002/mp.17985 40665561 PMC12694766

[46] Yalamanchili A, Sengupta B, Song J, Lim S, Thomas TO, Mittal BB, et al. Quality of Large Language Model Responses to Radiation Oncology Patient Care Questions. JAMA Netw Open. 2024;7(4):e244630. doi: 10.1001/jamanetworkopen.2024.4630 38564215 PMC10988356

[47] Hao Y, Holmes J, Hobson J, Bennett A, McKone EL, Ebner DK, et al. Retrospective Comparative Analysis of Prostate Cancer In-Basket Messages: Responses From Closed-Domain Large Language Models Versus Clinical Teams. Mayo Clin Proc Digit Health. 2025;3(1):100198. doi: 10.1016/j.mcpdig.2025.100198 40130001 PMC11932704

[48] Holmes J, Liu Z, Zhang L, Ding Y, Sio TT, McGee LA, et al. Evaluating large language models on a highly-specialized topic, radiation oncology physics. Front Oncol. 2023;13:1219326. doi: 10.3389/fonc.2023.1219326 37529688 PMC10388568

[49] Zhang QS, Kang J, Lybarger K, Glenn MC, Sponseller P, Blau KH, et al. Semi-automated topic identification for radiation oncology safety event reports using natural language processing and statistical modeling. Med Phys. 2025;52(8):e17936. doi: 10.1002/mp.17936 40781843

[50] Beidler P, Nguyen M, Lybarger K, Holmberg O, Ford E, Kang J. Automated Triaging and Transfer Learning of Incident Learning Safety Reports Using Large Language Representational Models. arXiv preprint arXiv:250913706. 2025.

[51] Yuan W, Xu R, Peng S, Guo Y. Large language models in cardiovascular imaging: current applications and future prospects. Med Research. 2025.

[52] Yang B, Kuai F, Chen Z, Fu D, Liu J, Wu Y, et al. miR-634 Decreases the Radioresistance of Human Breast Cancer Cells by Targeting STAT3. Cancer Biother Radiopharm. 2020;35(3):241–8. doi: 10.1089/cbr.2019.3220 32077744

[53] Yang S, Han G, Chen Q, Yu L, Wang P, Zhang Q, et al. Au-Pt Nanoparticle Formulation as a Radiosensitizer for Radiotherapy with Dual Effects. Int J Nanomedicine. 2021;16:239–48. doi: 10.2147/IJN.S287523 33469284 PMC7811476

[54] Thomadsen B, Kapur A, Blankenship B, Caldwell B, Claps L, Cunningham J. AAPM task group report 288: Recommendations for guiding radiotherapy event narratives. Medical Physics. 2024;51(9):5858–72. 10.1002/mp.17282 39073127

[55] Huq MS, Fraass BA, Dunscombe PB, Gibbons JP, Ibbott GS, Mundt AJ. The report of Task Group 100 of the AAPM: Application of risk analysis methods to radiation therapy quality management. Medical Physics. 2016;43(7):4209–62. 10.1118/1.4947547 PMC4985013 27370140

[56] Wang Y, Najad-Davarani SP, Bossart E, Studenski MT, De Ornelas M, Yang Y. Quantitative Risk Assessment in Radiation Oncology via LLM-Powered Root Cause Analysis of Incident Reports. 2025. doi: arXiv:251102223

